# Observation of Transient Prenucleation Species of Calcium Carbonate by DNP-Enhanced NMR

**DOI:** 10.1021/acs.jpclett.4c01588

**Published:** 2024-07-29

**Authors:** Martins Balodis, Yu Rao, Gabriele Stevanato, Matthias Kellner, Josephine Meibom, Mattia Negroni, Bradley F. Chmelka, Lyndon Emsley

**Affiliations:** † Institut des Sciences et Ingénierie Chimiques, École Polytechnique Fédérale de Lausanne (EPFL), CH-1015 Lausanne, Switzerland; ‡ Department of Chemical Engineering, University of California, Santa Barbara, California 93106-5080, United States

## Abstract

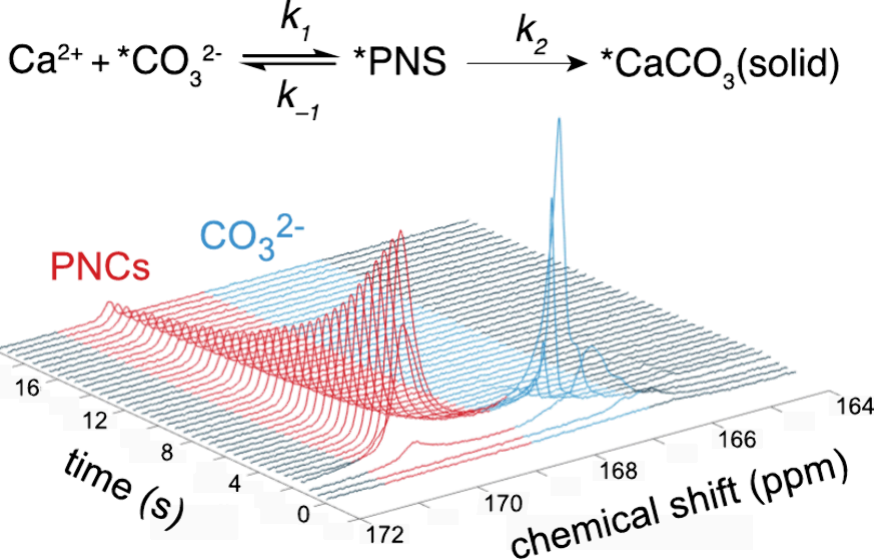

Knowledge of the mechanism by which polymorphic inorganic species, such as carbonates, are formed is crucial to understand and guide the selective crystallization of end products. Recently it has been shown that a key step in the crystallization of calcium carbonate is the formation of intermediate species known as prenucleation clusters. However, the observation of these prenucleation clusters in solution is exceedingly challenging because of their short lifetime and low concentrations. Here, using dissolution DNP-enhanced NMR spectroscopy, we observe signals from prenucleation species of calcium carbonate from which the kinetics of formation and conversion are determined.

Formation of calcium carbonate (CaCO_3_) is of great importance in biomineralization, geosciences, and industry. CaCO_3_ is one of the most prevalent minerals in the Earth’s crust, and it strongly affects the chemistry of ocean water.^1^ CaCO_3_ is a primary precursor for cements (4 billion tonnes/year), and it is used in industrial applications, such as a filler for polymers or in paper processing to modify their mechanical properties or coloration.^2^ The development of methods for CO_2_ capture for conversion back to CaCO_3_, typically via reactions and precipitation of solid products from solution, is a subject of high current interest for climate change abatement.^3,4^ To conduct such multiphase reactions at large scale requires continuous processes that are difficult to control with respect to the rates of reaction, diffusion and mixing, and product selectivity.^5^ Therefore, understanding the mechanisms underlying the formation of CaCO_3_ is of key importance, as it would guide the development of approaches to the controlled formation of different polymorphs, particle sizes, and morphologies.

The known crystalline phases of CaCO_3_ are calcite, aragonite, and vaterite.^6^ Two hydrated phases of CaCO_3_, the monohydrate (CaCO_3_·H_2_O) and hexahydrate (CaCO_3_·6H_2_O), are also known. In addition, amorphous calcium carbonate (ACC), which normally exists as a monohydrate,^7^ is a source for the development of calcium carbonate-based shells and can be a precursor of the crystalline phases.^8−10^ Although the main reactions^8^ leading to the formation of CaCO_3_ look simple,
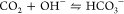
1
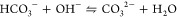
2
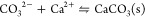
3the nucleation processes that lead to solid CaCO_3_ products are complicated and not fully understood even after 100 years of research.^11^ Nucleation is the local emergence of a distinct thermodynamic phase at the nanoscale that then grows to form a macroscopic solid. The phase changes involved in nucleation happen on a length scale of 10^–10^ m, in mixtures with concentrations in the nM–mM range, and time scales shorter than 1 s, thus rendering studies of nucleation very challenging.^12^ Recently, so-called prenucleation clusters (PNCs) were observed in the formation of CaCO_3_ (Figure 1) by using calcium ion potential measurements.^11^ This challenged the classical nucleation and growth theory (CNT) that has been developed to describe the formation of solid CaCO_3_.^13^ Classical growth theory proposes that the formation of crystals proceeds via spontaneous production of small crystalline nuclei that coalesce to form larger crystals when the solution is supersaturated with respect to the crystalline CaCO_3_. Usually, however, amorphous calcium carbonate forms before the crystalline phase, and there are a few proposed mechanisms to explain how that happens. One is via chain-like ion clusters, referred to as prenucleation clusters.^11,14,15^ A second is via a separate water phase that is rich in Ca^2+^ and CO_3_^2–^ ions and dehydrates to form ACC.^16−19^ Finally, third is via the formation of small amorphous particles that grow larger in size and then either directly or indirectly (through crystalline nuclei) convert to crystalline CaCO_3_.^20^ These three different types of CaCO_3_ aggregates are usually globally termed as prenucleation species (PNS).^21^ While there is now intense discussion concerning the mechanism of CaCO_3_ crystal growth,^22−27,23,28−31^ it is evident that the formation and the subsequent transformation of PNS are intimately connected to the formation of the final stable phase, as has been established by following the slow evolution of prenucleation species in the CaP crystallization process.^32^

**Figure 1:**
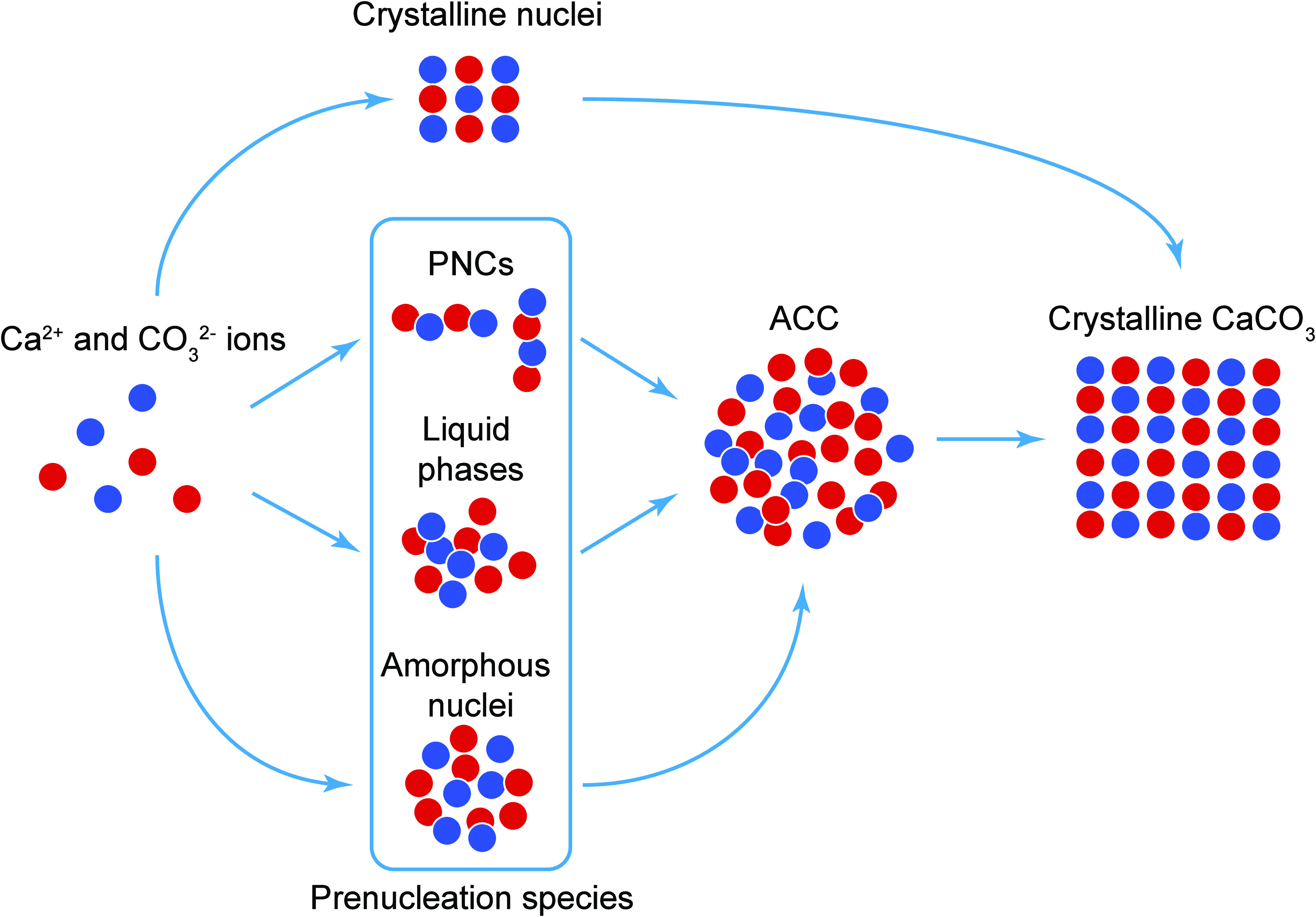
Simplified scheme of proposed mechanisms of calcium carbonate crystal formation.^33^

Direct observation of the PNS, however, is exceedingly challenging due to their dilute concentrations, nanoscale dimensions, absence of long-range order, and transient conditions. Calcium carbonate PNS were first observed by ion potential measurements combined with analytical centrifugation,^11^ and later this observation was corroborated by cryo-TEM.^15^ Solution clusters have also been shown for other species. Solatini et al. observed l-(+)-tartaric acid solution clusters formed via analytical ultracentrifugation combined with terahertz spectroscopy.^34^ However, the exact nature of the composition and structure of these types of species so far has been elusive.

NMR spectroscopy would be ideally placed to provide detailed insight into the species formed during nucleation and their kinetics. Indeed, NMR has been employed to characterize different aspects of the crystallization process.^35^ However, these transient species are present at low concentrations that are infeasible to analyze by conventional NMR techniques. Nevertheless, dynamic nuclear polarization (DNP) has been developed as a technique to significantly enhance NMR signals and can be used to analyze species present at even very low concentrations.^36^ DNP in solids has been used to observe the crystallization process, but requires quenching the reaction at low temperature.^37^ Here, we use a dissolution DNP NMR (dDNP) scheme to detect and identify transient carbonate species that are formed during the first few seconds of nucleation. The use of dDNP allows the real-time observation of the transient species generated in the first few seconds of the nucleation process by amplifying the ^13^C NMR signal by a factor >1000. We observe signals that we attribute to PNS and directly monitor their nucleation and growth kinetics in solution from free carbonate ions and their subsequent conversion to solid CaCO_3_ products.

Several methods have been used to characterize the PNS of calcium salts, including titration,^11^ small-angle X-ray scattering,^38^ electron microscopy,^15^ THz spectroscopy,^18^ and energy dispersive X-ray spectroscopy.^32^ NMR is of particular interest since it would allow direct observation of PNS and could distinguish between mixtures of species due to the resolution provided by chemical shifts. However, NMR of PNS is challenging because of the low concentration, intrinsic low sensitivity and the short lifetime of the PNS. Indeed, the transformation of PNS into CaCO_3_ has, until now, not been observed by NMR in solution or suspensions. ^31^P NMR has been employed to follow long-lived PNS of calcium phosphate in simulated body fluids.^32^ Calcium phosphate has also been studied by dissolution DNP-NMR,^21^ where Weber et al.^21^ showed that it is possible to monitor the PNS formation in real-time by directly mixing in the spectrometer solutions of CaCl_2_ and hyperpolarized phosphate solutions.^39^

Here, we use a similar approach to directly hyperpolarize sodium carbonate and observe the formation of PNS in the ^13^C NMR spectra. To that end, we hyperpolarized frozen ^13^C-labeled sodium carbonate solutions at 1.4 K (Figure 2). Once polarized, the sample was rapidly melted by contact and mixing with a superheated buffer, after which the resulting solution was transferred to a 11.7 T NMR magnet, where it was mixed with an aqueous solution of calcium chloride waiting in the NMR tube (Figure 2), at which point the reaction was initiated. We note that, under the basic conditions used here, the formation of CO_2_ during the dissolution process from Na_2_CO_3_ is expected to be insignificant. Also, any CO_2_ that was formed during dissolution will be retransformed into bicarbonate/carbonate. We also note that the presence of ∼1% glycerol (required as glass forming agent for DNP) and 1 mM polarizing agent TEMPOL in the nucleating solution have previously been shown not to perturb the precipitation pathway of CaCO_3_, even at much higher concentrations than used here.^40^ Under these conditions, the resulting calcium carbonate finally crystallizes to calcite, as confirmed by solid-state NMR in Figure S6. The precise composition of the different solutions is described in Methodsof the Supporting Information.

**Figure 2:**
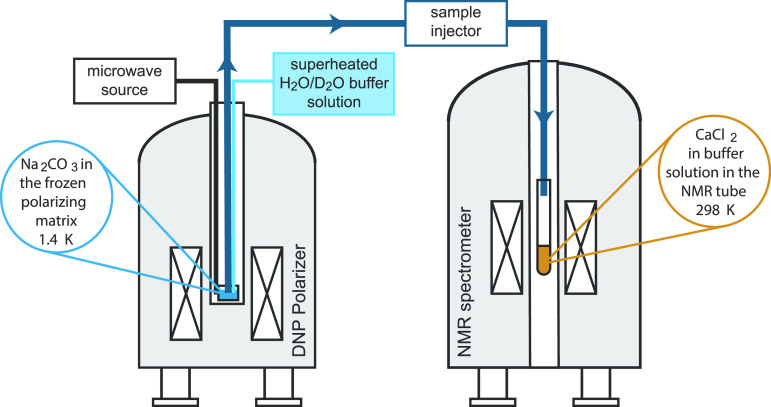
Schematic diagram showing the main components of the experimental setup. A 100 μL aliquot of 1 M ^13^C-labeled Na_2_CO_3_ solution in 10:30:60 H_2_O:D_2_O:glycerol-*d*_8_ containing 50 mM TEMPOL radical is inserted into the DNP polarizer and directly polarized for 2–3 h. After the polarization step, melting and dissolution are achieved by adding 5 mL of superheated buffer solution to the sample in the polarizer, flash dissolving it, and transferring the hyperpolarized solution to a conventional NMR tube in the NMR spectrometer. The NMR tube contained 50 μL of a buffer solution and a variable amount of dissolved CaCl_2_. The solution at the moment just after mixing contained 20 mM Na_2_CO_3_ and 10, 20, or 40 mM CaCl_2_, before conversion to CaCO_3_.

To follow the evolution of the carbonate species after mixing, a series of 1D ^13^C NMR spectra were acquired, each with a single scan after a 30° pulse, every 0.5 s until the magnetization had completely decayed. Using the parameters described in Methodsin the Supporting Information, we were able to obtain ^13^C polarization levels of around 30% for the ^13^C nuclear spins in the frozen Na_2_CO_3_ solutions. This is a factor 30000 higher than the thermal polarization of ^13^C nuclear spins at room temperature. Even when taking into account losses incurred during the dissolution and transfer process, which here typically took a total of 10 s, this very significant enhancement is what enables the detectability of the ^13^C signals from transient intermediates in a single scan that would otherwise be well below the detection limit of NMR spectroscopy.

Four examples of ^13^C spectra evolving over time are shown in Figure 3 at buffer pH values of 10 and 11 and with different calcium-to-carbonate ratios of 2:1 and 1:1. Following dissolution and transfer from the polarizer to the NMR spectrometer, the hyperpolarized carbonate solutions are injected into an NMR tube which contains the calcium chloride solution. During the initial mixing process there is a volume change and some turbulence in the solution which influences peak shapes and the chemical shifts. As a result, any transient species that only lasts for <2 s after mixing will be invisible. Improvement of the hardware, in particular the injection system, would be required to shorten this blind window. After 1–2 s, the solution settles and the NMR response stabilizes. Under these ^13^C dDNP-NMR conditions, we measured the enhancement to be 1600, which is consistent with the signal-to-noise ratio of >50 in the first few seconds of our target signals. In the typical ^13^C chemical shift region of carbonate species (164–170 ppm) for Figure 3a with the buffer pH = 11 and a calcium-to-carbonate ratio of 2:1, we observe a set of ^13^C signals at 168–170 ppm and another set of peaks at <168 ppm. To assign these two regions of intensity, we performed a thermal experiment without Ca^2+^ while keeping the rest of the composition the same (Figure S1), for which a peak associated with soluble carbonate–-bicarbonate anions is observed at 167.5 ppm. Therefore, we can assign the set of peaks near 167.5 ppm in the dissolution DNP-NMR spectra to solvated carbonate ions. The reason we do not see a single sharp peak, but instead a broader asymmetric distribution of intensity, is due to the fact the mixture is not homogeneously mixed immediately after mixing; a solid phase is rapidly formed and is unequally distributed in the NMR tube, leading to a range of slightly different local solution environments. The intensity at higher frequency manifests a behavior that we attribute to PNS, appearing at a higher isotropic ^13^C chemical shift due to the effects of electrostatic interactions with calcium ions. The intensity of the peak associated with free carbonate anions decreases with time, consistent with their depletion as they are consumed to form the PNS (along with contributions from the loss of hyperpolarized signal due to the 30° pulses used for detection and spin relaxation.) In contrast, the ^13^C signal from the PNS species appears rapidly, as PNS are formed from the free carbonate and then decay due to loss to precipitation, the 30° pulses used for detection, and spin relaxation. (Notably, only the part of the solution which is inside the coil is depolarized by any given pulse, which makes an apparent decay slower than that expected from 30° pulses due to convection in and out of the coil region.) Figure 3b shows a spectrum under conditions identical to those in Figure 3a to provide an idea of the robustness and reproducibility of the experiments and the subsequent analysis. While the PNS peaks are differently shaped due to inhomogeneity in the solution, The ^13^C intensity from the PNS species is centered at the same chemical shift as in Figure 3a and with similar decay behavior, though interestingly exhibiting line shape differences that manifest different extents of inhomogeneous mixing to which the measurements are sensitive.

**Figure 3:**
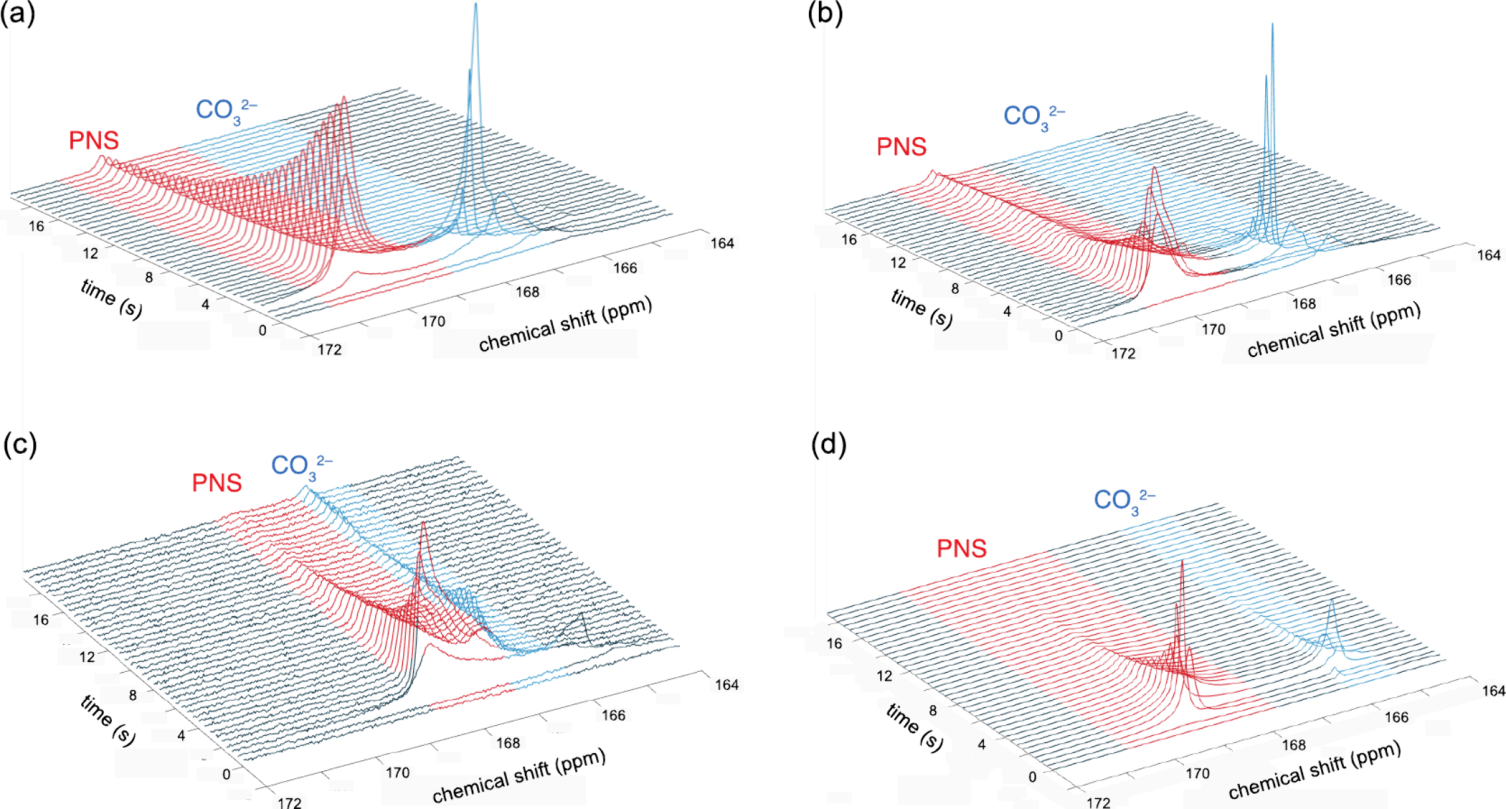
Single-scan ^13^C NMR spectra acquired at 11.7 T at room temperature, as a function of time after the dissolution and mixing of aqueous CaCl_2_ and hyperpolarized Na_2_CO_3_ solutions. Spectra were acquired every 0.5 s with a 30° pulse, and spectra are shown for 20 s after the solutions were mixed with the following compositions: (a) pH = 11, calcium to carbonate ratio of 2:1; (b) repetition of panel a; (c) pH = 11, calcium to carbonate ratio of 1:1; (d) pH = 10, calcium to carbonate ratio of 2:1. The ^13^C chemical shift ranges for free carbonate ions and for the PNS are shown in blue and red, respectively.

Figure 3c shows the ^13^C dDNP-NMR spectrum obtained when the ratio of calcium to carbonate is changed to 1:1, while maintaining the pH = 11. We find a qualitatively similar behavior but with some very significant differences. First, the isotropic ^13^C chemical shift of the PNS peaks at the lower calcium concentration is 2 ppm lower, which suggests that the PNS do not have the same composition. Our hypothesis is that the composition of PNS formed with excess Ca^2+^ contains more Ca^2+^ which yields a higher chemical shift of the ^13^C signal because of the stronger interaction of the carbonate species in the PNS with Ca^2+^. Second, the signal of PNS also lasts beyond 20 s for the case of 2 Ca:1 C, whereas it has completely decayed after about 10 s for the case of 1 Ca:1 C. This is unlikely to be caused by the generation of PNS from the free carbonates, as the carbonate peak vanished at this stage. Here, we attribute the long lifetime of PNS in the 2:1 case to a slower forward transformation to bulk ACC. Combining these two observations, we propose that the high Ca^2+^ composition might stabilize the PNS and prevent further transformation to ACC. Compared to the PNS, the free carbonate peaks also show a similar but less marked chemical shift change with the different calcium- to-carbonate ratios. Consequently, the two peaks are less separated in the carbonate-abundant environment. Unsurprisingly, the free carbonate peak survives longer as the calcium concentration is lowered as the consumption of carbonate is proportionally reduced.

It is worth mentioning that we observe the formation of two carbonate–glycerol esters which form during the dissolution process when the temperature is high (Scheme S1), but that does not occur in the reaction mixture at room temperature.^41^ Therefore, this side reaction reduces the effective starting carbonate concentration but is not expected to interfere with the nucleation reaction we are monitoring. The two ^13^C peaks corresponding to these byproducts are located at 160.2 and 160.7 ppm, respectively, which are out of the spectral range shown in Figure 3 (see Figure S2).

Figure 3d shows the ^13^C dDNP-NMR spectrum obtained using a buffer pH = 10 with a calcium-to-carbonate ratio of 2:1. The spectrum is very similar to that acquired at pH = 11 (Figure 3a), though with an important difference: a weak ^13^C signal is observed at 165.9 ppm that persists for over 10 s and is assigned to a byproduct formed from a reaction of the alanine in the buffer solution with the carbonate during the dissolution process, as shown in Scheme S2.^42^ The other byproducts from carbonate and glycerol mentioned above are still present.

The strong transient^13^C peak at 165 ppm is assigned to hyperpolarized solvated carbonate anions which is only observed for 2–3 s, and which again indicates rapid consumption of carbonate in the Ca^2+^-abundant conditions. Compared to pH = 11, signal from the free carbonate species appears at a lower chemical shift, which suggests the carbonate/bicarbonate equilibrium is shifted toward bicarbonate. The peak corresponding to PNS is observed at 169 ppm, which is also decreased compared to pH = 11. This lower chemical shift value suggests that there is also a relatively higher concentration of bicarbonate in the PNS.

The qualitative analysis of the transient signals observed in the ^13^C dDNP-NMR spectra described above is already highly informative. However, the integrals of the different peaks as a function of time should allow for an estimation of the reaction rates that contribute to the nucleation process. To demonstrate such an estimation of the kinetics, we analyze the data here using the chemical reaction scheme of Figure 4 as a model. The overall reaction is divided into two steps: first, the formation of PNS and then the subsequent transformation of the PNS into solid CaCO_3_, which is not visible in solution-state ^13^C NMR spectra. The formation of the PNS is in principle a dynamic equilibrium, whereas the formation of precipitated CaCO_3_ is here approximated to be irreversible. Each carbonate species is composed of a hyperpolarized and an unpolarized (or negligibly) polarized population, with the hyperpolarized species relaxing to the unpolarized form with the corresponding ^13^C nuclear spin–lattice relaxation time *T*_1_. The change in concentrations (regardless of the polarization) due to the reactions in Figure 4 can be written
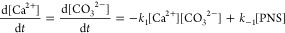
4
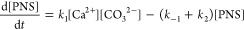
5The integrated intensities of the ^13^C NMR signals are proportional to the magnetization of the corresponding species, which in turn is proportional to the concentration of the polarized species with a uniform scaling factor, *M*:
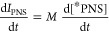
6
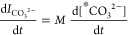
7The rates at which the polarized species are transformed into unpolarized species depends on both their respective ^13^C *T*_1_ values and on the series of pulses applied for observation. To simplify the calculation, we use the same *T*_1_ value here for all species (since the *T*_1_s are in any case here much slower than any of the other rates). Ideally, the decay caused by the 30° pulse is 13.4% (1 – cos(30°)) per 0.5 s, but as discussed above, the convection of species in and out of the coil region will significantly reduce the apparent rates of signal decay. Therefore, we define an effective pulse decay rate *R*_e_ that is slower than the predicted pulse decay rate. Taking these factors into account, the complete description of the dynamics of these species is modeled as
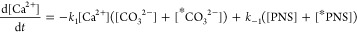
8
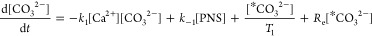
9
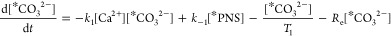
10
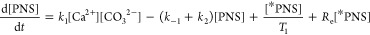
11
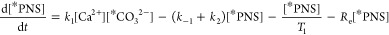
12Note that because the extent of hyperpolarization is so large and the thermal signal is negligible by comparison, it is reasonable to treat all of the decay rates as if they reduce the magnetization ultimately to zero. To fit the experimental data, we assume that the initial concentrations of PNS (both polarized and nonpolarized) are zero. The initial concentration of calcium used in the experiments is known, as is the initial total concentration of carbonate species, at least in principle. However, considering the consumption of free carbonates by the side reaction discussed above, we reduced the initial concentration accordingly. The exact ratio of the polarized to nonpolarized carbonate species depends on the degree of ^13^C signal enhancement, which is only known approximately (∼30%). Nevertheless, if the contribution of thermal polarization is neglected; then this ratio can be included in the scaling factor, which is an adjustable parameter of the fit. We then fit the data by numerical least-squares fitting between the predictions and the data. Because of the turbulent mixing that occurs during the first few seconds, we excluded the first few points from the fit. In addition, as the dynamic behavior is mainly observed at early times of the experiment, we fit the data over 8 s of the reaction, following the excluded initial points. Full details, and the code used, together with the numerical values of the fits, are given in the Supporting Information.

**Figure 4:**
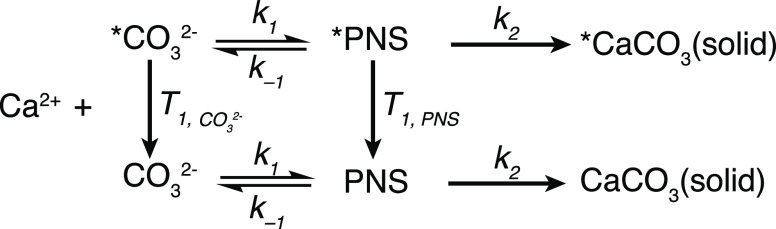
Reaction scheme and spin relaxation pathways used here to model the transient peak intensities observed in the ^13^C dDNP-NMR spectra. Asterisks indicate hyperpolarized species.

The results of the fits and the kinetic parameters that are determined from the ^13^C dDNP-NMR spectra in Figure 3 are shown in Figure 5. The longer lifetime of the carbonate ^13^C signal in the presence of a decreased calcium concentration is clearly reproduced. For the three experiments shown that were performed in the pH = 11 buffer solutions, the fitted kinetic properties are quite similar. Both the rate coefficients determined for the formation of the PNS, *k*_1_, and the forward transformation of PNS into solid CaCO_3_, *k*_2_, are similar in the three fits. The former changes in the range 20–31 M^–1^ s^–1^ and the latter fluctuates by less than 10% around 0.27 s^–1^. The values determined for the other consumption pathways for the PNS, *k*_*–*1_, vary in a range between 0.02 and 0.09 s^–1^. The estimated errors for the kinetic parameters (shown in Figure 5) are calculated using a Monte Carlo analysis based on random noise in the measured integrals of 0.025. Using this estimate, the variations in the measured rate coefficients between the different samples are all within the errors. However, we remark that there are significant deviations from the model behavior that could lead to larger errors.

**Figure 5:**
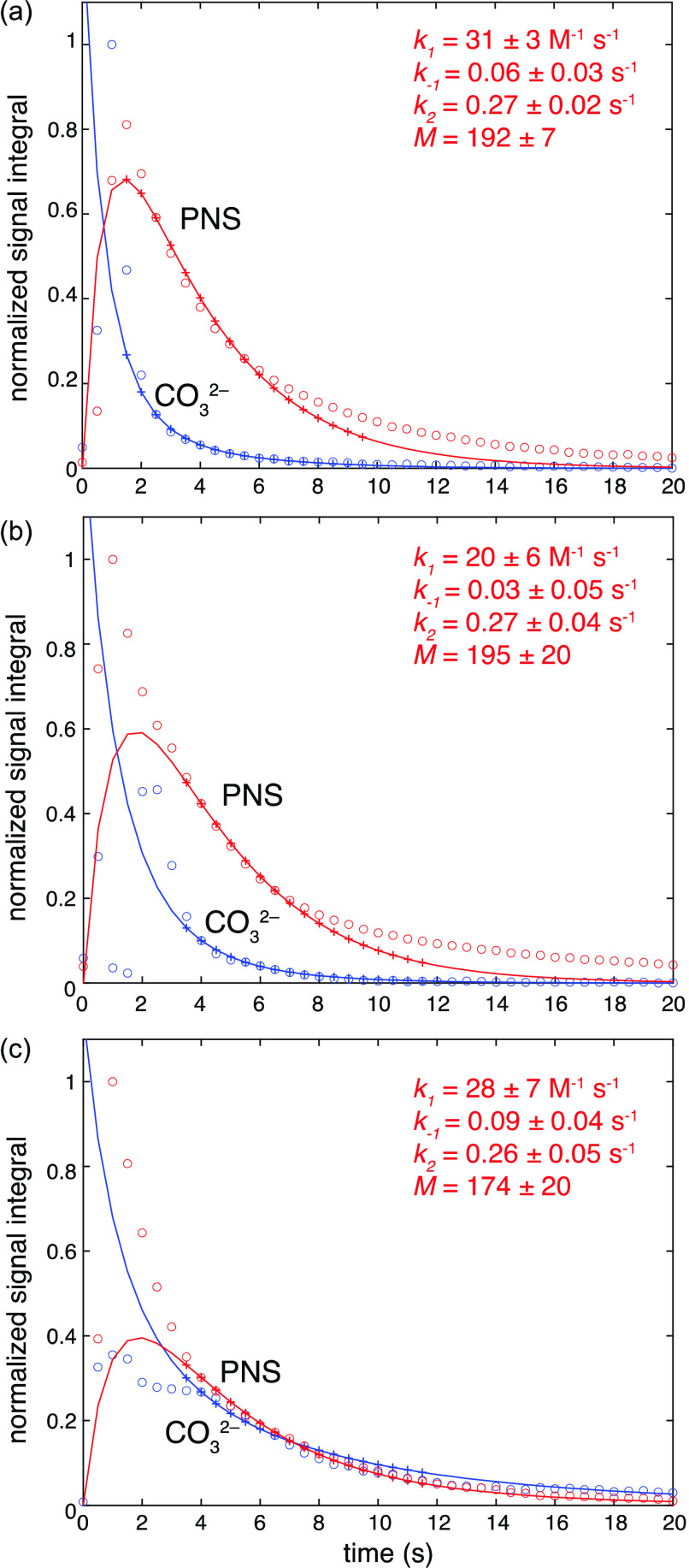
Experimental data (open circles) for the evolution of NMR signal integrals of the carbonate (blue) and PNS (red) signals, together with the parameters that yield the best fits and the resulting fits, which are shown as solid lines and crosses in the region that was used for the fit. (a–c) Three data sets shown in Figure 3, with pH = 11 and (a) calcium to carbonate ratio of 2:1, (b) repetition of panel a, and (c) calcium to carbonate ratio of 1:1. The estimated errors on the fitted values were calculated assuming a random noise level of the integrals of ±0.025, as described in the Supporting Information.

Overall, as expected, considering the high concentration of calcium and carbonate present in the early stages, we observe that the formation of PNS from free carbonate is first dominant and is then gradually replaced by the forward transformation of PNS to the solid calcium carbonate since *k*_2_ is always significantly larger than *k*_*–*1_. Therefore, despite the potential sources of error, the two dominant reactions in the system are clearly characterized in the fits.

This analysis demonstrates the potential of dissolution DNP-NMR for real-time kinetic measurements to quantify the reaction rates of short-lived solution species. It should be pointed out that in these experiments there are likely several considerations that increase the uncertainty of the values so that the results should be considered semiquantitative. These include systematic deviations from the data, which we attribute to inhomogeneous mixing and settling at the very early times and to the effects of convection at longer times. For example, we found that the fit usually yields a decay rate faster than the experimental data for the PNS signals beyond 8 s, which might be attributed to a convection effect that is difficult to model. All of these factors can potentially be reduced by improving the experimental configuration and the kinetic model used to provide more accurate measurements of the kinetic parameters.

In conclusion, transient species formed during the first few seconds of the formation of calcium carbonate from solution have been observed, and their rates of formation or disappearance have been quantified by using high-field ^13^C dissolution DNP-NMR spectroscopy. Signals were observed that are attributed to PNS, and the time dependence of the signals was used to directly monitor the kinetics of creation of PNS in solution from free carbonate ions and their subsequent conversion into the solid form. The observation of PNS was enabled by the hyperpolarization at 1.4 K of frozen ^13^C-labeled sodium carbonate solutions, which were then melted and mixed rapidly with aqueous calcium chloride solutions waiting in the high-field NMR spectrometer, at which point the reaction starts. We estimate ^13^C signal enhancement factors > 1000, a significant enhancement that enables the detectability of the ^13^C signals from transient intermediates in a single scan, which would otherwise be well below the detection limit of NMR spectroscopy. Estimated values for reaction rate coefficients for the formation and consumption of the PNS were obtained by fitting the data to a model for the prenucleation process. We note that we do not characterize the detailed structures of the PNS observed here, and modeling and interpreting the measured chemical shifts in terms of potential structures present will be the subject of future studies. The approaches developed here should also be important in the future for measuring near-nucleation phenomena in other carbonate-based materials.

## Supporting Information Available

Experimental details, supplementary figures and tables, details of the fitting procedure, and a link to all of the raw data (PDF)
