# Supplementary material for: Observation of Transient Prenucleation Species of Calcium Carbonate by DNP-Enhanced NMR
Source: J Phys Chem Lett. 2024 Jul 29;15(31):7954–61. doi: 10.1021/acs.jpclett.4c01588 (PMC11318035; doi:10.1021/acs.jpclett.4c01588)
Supplement: Supplementary file 1 — jz4c01588_si_001.pdf [file jz4c01588_si_001.pdf]

Supporting Information for

Observation of Transient Prenucleation Species of Calcium Carbonate by DNP Enhanced NMR

Martins Balodis[1], Yu Rao[1], Gabriele Stevanato[1], Matthias Kellner[1], Josephine Meibom[1], Mattia Negroni[1], Bradley F. Chmelka[2] and Lyndon Emsley[1]*

[1]Institut des Sciences et Ingénierie Chimiques, École Polytechnique Fédérale de Lausanne (EPFL), CH-1015 Lausanne, Switzerland

[2]Department of Chemical Engineering, University of California, Santa Barbara, California 93106-5080, U.S.A.

AUTHOR INFORMATION

Corresponding Author

*email: lyndon.emsley@epfl.ch

Table of Contents

Raw Data Statement	S1
Methods	S2
Details of the fitting procedure	S4

Raw Data:

The NMR raw data is available [link will be added after acceptance] in the original TopSpin, JCAMP formats, together with the MATLAB fitting codes used to determine the kinetic parameters. Data are made available under the license CC-BY-4.0 (http://creativecommons.org/licenses/by/4.0/

Methods:

Dissolution DNP experiments were carried out on a home-built 6.7 T polarizer described previously. 100 μL of a 1M solution of carbon-13 labelled Na_2CO_3 (Sigma Aldrich) dissolved in H_2O: D_2O: glycerol-d_8 = 1: 3: 6, and containing 50 mM TEMPOL radical as the hyperpolarization agent, was loaded into a sample holder made from PEEK (polyether ether ketone). The sample holder was placed into the cryostat and ^{13}C nuclei were directly polarised using microwave irradiation for 2–3 hours while simultaneously following the growth of hyperpolarization polarisation by applying 1° pulses every 5 minutes. To access higher polarisation the temperature was cooled to 1.4K by lowering the

internal pressure. Microwaves were provided by the Elva VCOM-10/0.5/94/400 microwave source at an output power of 400 mW. The microwave frequency was 188.250 MHz. Prior to dissolution 5 mL of an H_2O solution containing buffer (10.0 or 11.0 pH) and D_2O in 1:1 v/v proportion was superheated in a Teflon holder until the internal vapour pressure reached 10 bar. (The commercial buffer solutions used were a mixture of glycine, sodium chloride and sodium hydroxide (pH = 10.0) and diisopropylamine (pH = 11.0) (both from Mettler-Toledo). The superheated solution was injected to the sample holder, and then flushed to the NMR spectrometer via a magnetic tunnel. 750 μL of the hyperpolarized carbonate solution is injected into a standard 5 mm NMR tube in the detector which contains 50 μL of the buffer solution containing dissolved $CaCl_2$ with the calcium ion concentration being variable and in proportion to the final carbonate ion concentration (20 mM) in the solution. The proportions used were 1.0x and 2.0x (20 and 40 mM) of the carbonate ion concentration. The total sample is about 6 cm in the NMR tube while the coil covers about 2 cm of the liquid.

A pseudo 2D carbon-13 detection experiment was used to acquire the NMR spectra on a Bruker AVANCE NEO spectrometer operating at 11.7 T (125 MHz for ^{13}C) with a home-built probe. The temperatures of the samples at the time of acquisition were determined to be 21±1°C. 30° ^{13}C pulses were used with an acquisition time of 300 ms, and with a repetition delay between scans of 200 ms. The acquisition was started immediately before the dissolution, in order to follow the reaction from the very start. Glycerol (the glassing agent required for DNP) was present in all samples and used as an internal reference with the rightmost peak in the spectrum belonging to - CH_2OH groups set to 63.0 ppm. Spectra were processed with a line broadening of 1.5 Hz.

The dissolution DNP and NMR observation methods used here are similar to those used by Weber et al. for calcium phosphate,[1] with the replacement of phosphate (^{31}P NMR) by carbonate (^{13}C NMR). A main difference in our experiment is that the overheated solvent for dissolution is a mixture of D_2O and buffer solution instead of pure D_2O. With our protocol, the hyperpolarized species are thus mixed with buffer before the reaction, which is expected to reduce the fluctuation in pH after mixing. As the hyperpolarized solution contains the buffer solution, we no longer need a large volume of the calcium chloride solution, and the dilution due to mixing with calcium solution is therefore reduced (17:1 v:v dilution compared to 4:1 v:v dilution).

A scaled-up experiment was performed to collect and analyse the final product after crystallization. 14 mL pH=11 buffer solution of 0.02 M of carbon-13 labelled Na_2CO_3 with 1% glycerol and 1 mM of TEMPOL radical was used to mimic the final conditions in the dissolution DNP experiment. 2 equivalents of $CaCl_2$ were dissolved in D_2O and mixed with the solution mentioned above. The $CaCO_3$ solid that has formed after xx hours is then collected by centrifugation and packed into a 3.2 mm rotor for MAS NMR. The direct ^{13}C MAS NMR spectrum is then obtained via a spin echo pulse sequence on a commercial 9.4 T Bruker NMR spectrometer at room temperature at 8 kHz MAS, shown in Figure S6.

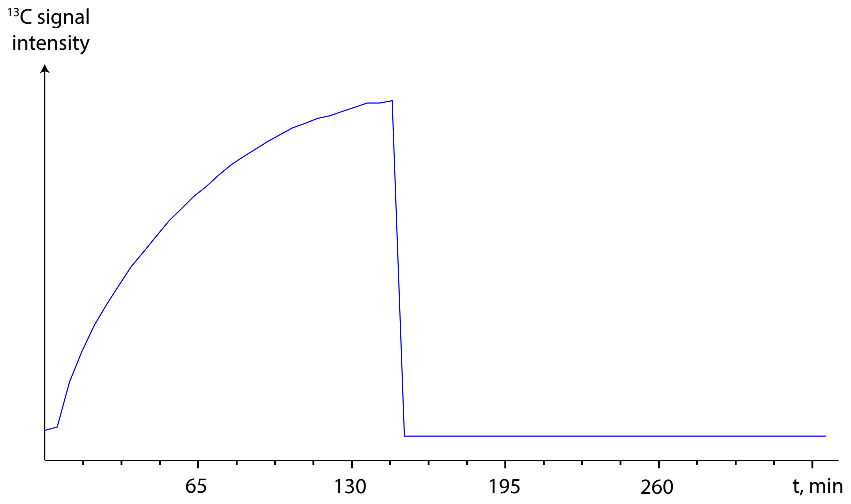

Figure S1. A typical hyperpolarization build-up curve of ^{13}C signal intensity associated with carbonate species in 1 M Na_2CO_3 in H_2O: D_2O: glycerol-d_8 = 1: 3: 6, and containing 50 mM TEMPOL, in the polarizer at 1.3 K. A plateau is reached after 2 hours of polarization. The curve is observed by applying a 1° pulse every 5 minutes.

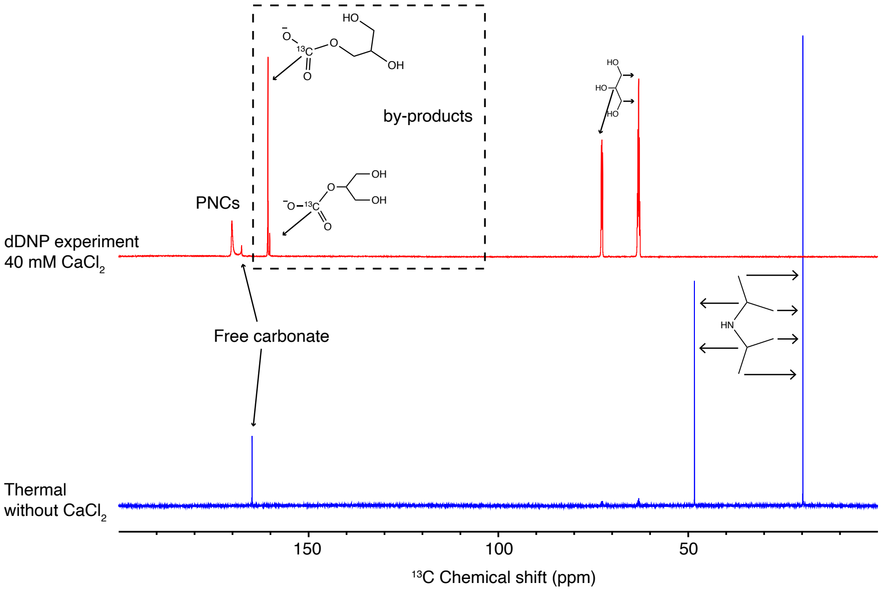

Figure S2. Single-pulse ^{13}C NMR spectra acquired at 11.7 T, (top, red) under DNP conditions (taken from the experiment in Figure 3a, hyperpolarized ^{13}C-enriched Na_2CO_3 with 40 mM of $CaCl_2$ in pH = 11 buffer and D_2O) and (bottom, blue) under conditions of thermal equilibrium (20 mM ^{13}C-enriched Na_2CO_3 in pH = 11 buffer and D_2O). Signals from the by-products formed from glycerol and carbonate and shown in the dashed box. In the DNP experiment, the glycerol peaks are observed because they are hyperpolarized. At thermal equilibrium, these signals are significantly weaker, and the two peaks from the buffer solution are observed.

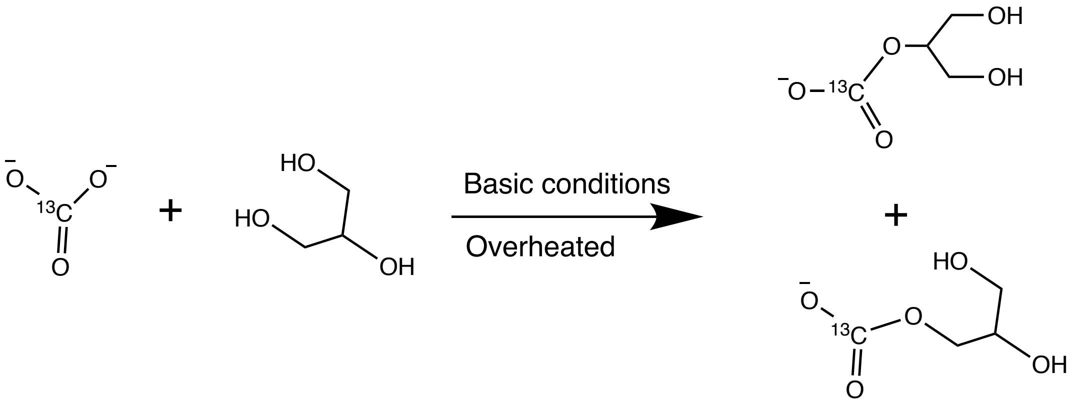

Scheme S1. The chemical reaction of the formation of the by-products from the reaction between carbonate and glycerol.

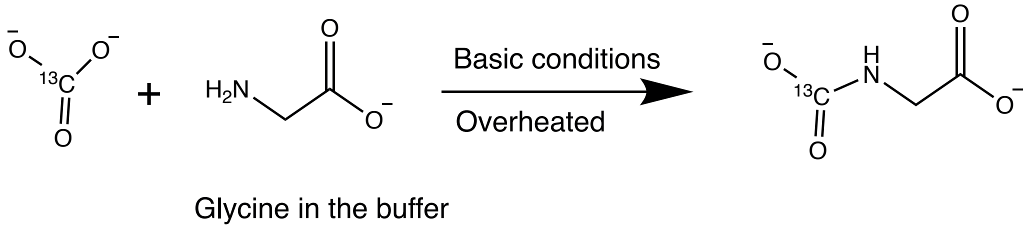

Scheme S2. The chemical reaction of the formation of the by-product in the pH= 10 experiment (Figure 3d).

$$\frac{d[Ca^{2+}]}{dt} = -k_1[Ca^{2+}]([CO_3^{2-}] + [^*CO_3^{2-}]) + k_{-1}([PNS] + [^*PNS]) \qquad \text{Eq. 1}$$

$$\frac{d[CO_3^{2-}]}{dt} = -k_1[Ca^{2+}][CO_3^{2-}] + k_{-1}[PNS] + \frac{[^*CO_3^{2-}]}{T_1} + R_e[^*CO_3^{2-}] \qquad \text{Eq. 2}$$

$$\frac{d[^*CO_3^{2-}]}{dt} = -k_1[Ca^{2+}][^*CO_3^{2-}] + k_{-1}[^*PNS] - \frac{[^*CO_3^{2-}]}{T_1} - R_e[^*CO_3^{2-}] \qquad \text{Eq. 3}$$

$$\frac{d[PNS]}{dt} = k_1[Ca^{2+}][CO_3^{2-}] - (k_{-1} + k_2)[PNS] + \frac{[^*PNS]}{T_1} + R_e[^*PNS] \qquad \text{Eq. 4}$$

$$\frac{d[^*PNS]}{dt} = k_1[Ca^{2+}][^*CO_3^{2-}] - (k_{-1} + k_2)[^*PNS] - \frac{[^*PNS]}{T_1} - R_e[^*PNS] \qquad \text{Eq. 5}$$

Details of the fitting procedure.

Using Equations 1–5, we calculated the evolution of each species and the NMR signal evolution during the observation time, and then fit this to the model parameters, using home written MATLAB codes (given together with the raw data at the link given above).

The initial concentrations of PNC species (both polarized and non-polarized) are zero. The initial total concentration of the carbonate species should be 0.02 M. Due to the consumption by the side reaction shown in Scheme S1, we reduced this concentration to 0.013 M. The exact ratio of the polarized to non-polarized carbonate depends on the exact enhancement, and is only known approximately (~30 %), but if we neglect the contribution of thermal polarization this ratio can be included in the scaling factor, which is a variable in the fit. Therefore, the initial non-polarized carbonate concentration is set to 0.09 M and the polarized carbonate concentration to 0.04 M. This polarization level does not play any important role in the fit as the overall scaling factor is a variable. The initial calcium concentration is 0.04 M or 0.02 M depending on the experiment. The T_1 of both PNCs and carbonates are set to 50 s. The effective relaxation rate caused by the pulse is set to 0.05 s^{-1}, which is lower than the theoretical value by the pulse (30 ° pulse per 0.5 second, equals 0.3 s^{-1})

The NMR signal integrals are linked to the concentration of the two polarized species by a scaling factor. To simplify the calculation, we normalized the integrals in each experiment. Together with the three reaction rate constants, we fit the four variables by changing these parameters to minimize the difference between the calculated and experimental results. The time point of 0 s (t = 0 s) is the same as in Figure 3, but certain numbers of points at the beginning are excluded from the fit due to the turbulence and volume change. Three points are excluded from (a), while seven points are excluded from (b) and (c). The 16 following points are then used in the fit, which are shown in as crosses in Figure 5.

To estimate the error of the fits, we used a Monte Carlo analysis where the best-fit data points are varied by ±0.025 and then refit to yield a distribution of fitted values, as shown in Figures S3-5. The errors shown in Figure 5 are one standard deviation measured for the distributions in Figures S3-5.

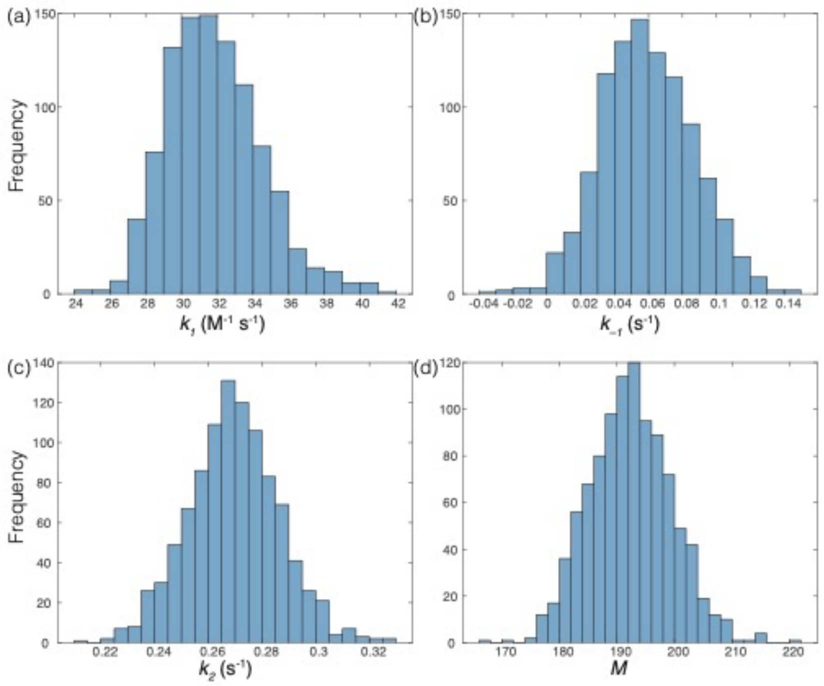

Figure S3. Distribution of fitted parameters for 1000 fits obtained as described in the SI text above for the data shown in Figure 5a.

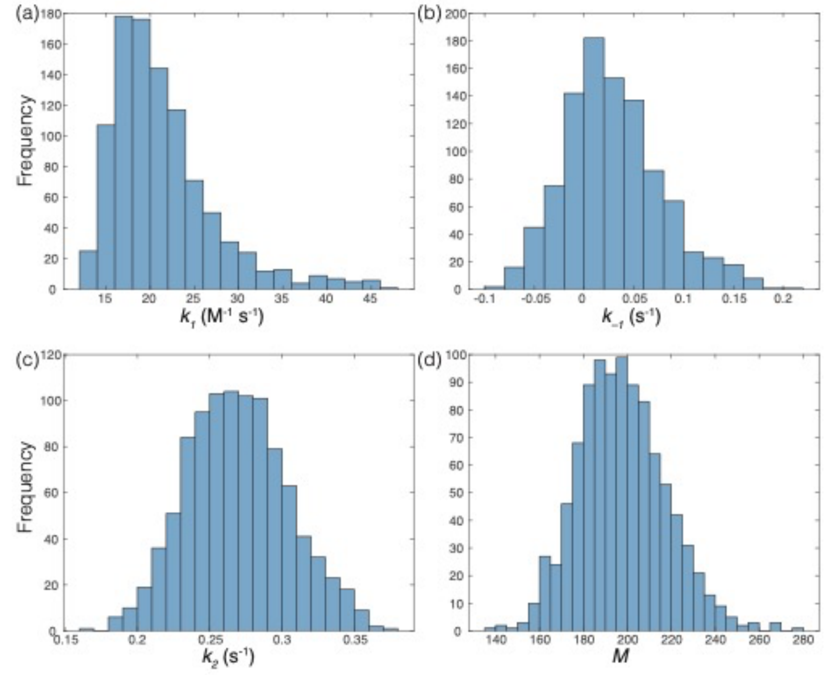

Figure S4. Distribution of fitted parameters for 1000 fits obtained as described in the SI text above for the data shown in Figure 5b. (Note that 20 outliers were removed corresponding to fits that did not converge).

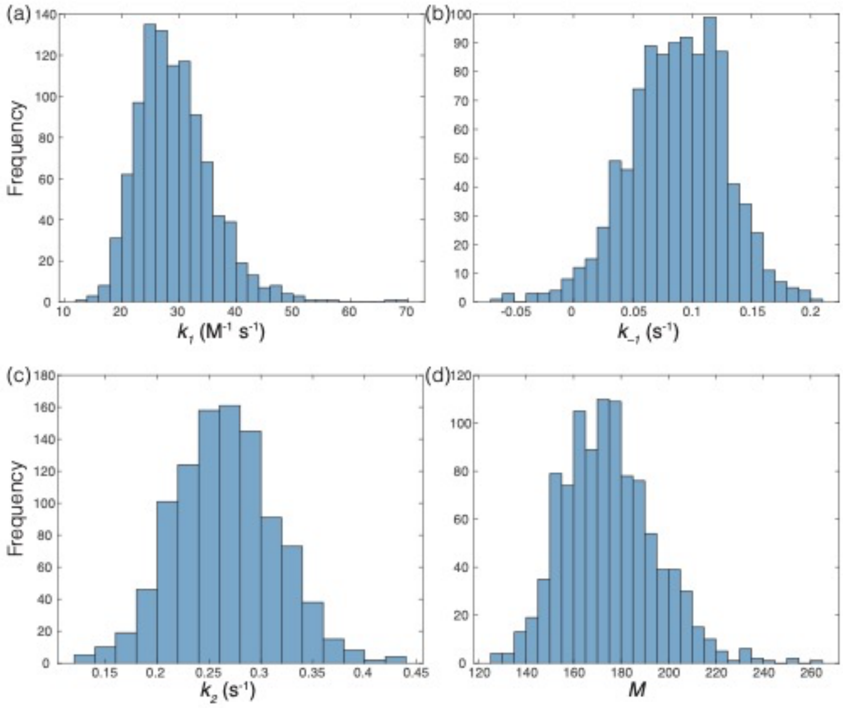

Figure S5. Distribution of fitted parameters for 1000 fits obtained as described in the SI text above for the data shown in Figure 5c.

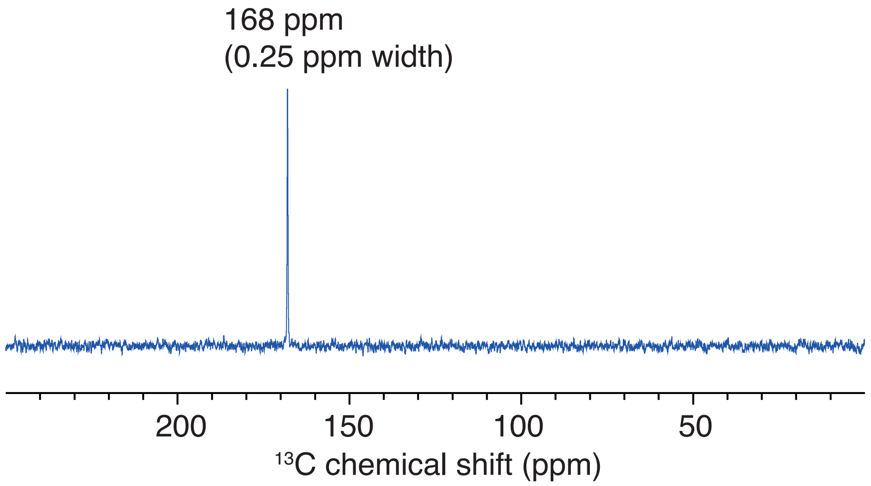

Figure S6. Directly detected ^{13}C MAS NMR spectrum of the solid $CaCO_3$ collected from the scaled-up experiment described in the methods section above. One peak is observed at 168 ppm with a narrow line width, which is consistent with calcite.[2]

References:

(1) Weber, E. M. M.; Kress, T.; Abergel, D.; Sewsurn, S.; Azaïs, T.; Kurzbach, D. Assessing the Onset of Calcium Phosphate Nucleation by Hyperpolarized Real-Time NMR. *Anal. Chem.* **2020**, *92*, 7666-7673.

(2) Nebel, H.; Neumann, M.; Mayer, C.; Epple, M. On the Structure of Amorphous Calcium Carbonates. A Detailed Study by Solid-State NMR Spectroscopy. *Inorg. Chem.* **2008**, *47*, 7874-7879.
